# Astragalus Polysaccharides/Chitosan Microspheres for Nasal Delivery: Preparation, Optimization, Characterization, and Pharmacodynamics

**DOI:** 10.3389/fphar.2020.00230

**Published:** 2020-03-18

**Authors:** Saisai Wang, Yuqing Sun, Jingjing Zhang, Xiaoming Cui, Zhilu Xu, Dejun Ding, Limin Zhao, Wentong Li, Weifen Zhang

**Affiliations:** ^1^School of Pharmacy, Weifang Medical University, Weifang, China; ^2^Department of Otolaryngology, Head and Neck Surgery, Central Hospital of Zibo, Zibo, China; ^3^College of Basic Medical, Qingdao Binhai University, Qingdao, China; ^4^Shandong Engineering Research Center for Smart Materials and Regenerative Medicine, Weifang Medical University, Weifang, China; ^5^Institute for Smart Materials and Regenerative Medicine, Weifang Medical University, Weifang, China; ^6^Department of Otolaryngology, Head and Neck Surgery, Affiliated Hospital of Weifang Medical University, Weifang, China; ^7^Department of Pathology, Weifang Medical University, Weifang, China

**Keywords:** Chitosan, Astragalus polysaccharide, spray drying, microspheres, nasal drug delivery

## Abstract

Chitosan (CTS) constitutes a promising area in treatment of nose-related diseases as a nasal drug delivery carrier. Astragalus polysaccharide (APS) significantly attenuates eosinophils and neutrophil-dominant airway inflammation, and it has a potential pharmaceutical application in the treatment of severe asthma. The purpose of this work was to prepare APS/CTS microspheres intended for nasal drug delivery by the spray-drying method. The characteristics of APS/CTS microspheres were evaluated by a scanning electron microscope, Fourier transform infrared spectroscopy, differential scanning calorimetry, and *in vitro* drug release. The effect of APS/CTS microspheres on rats with allergic rhinitis (AR) was investigated by eosinophil and neutrophil counts in nasal lavage fluid. Results of SEM showed that microspheres were spherical and wrinkled. *In vitro* release showed that 67.48–93.76% APS was released from APS/CTS microspheres at pH 6.8 within 24 h. The effects showed that APS/CTS microspheres alleviated allergic symptoms and reduced eosinophils infiltration and the expression of interleukin-4 in the nasal mucosa tissue of rats that had no liver and kidney toxicity by hematoxylin-eosin staining observation. In conclusion, these results indicated that APS/CTS microspheres had excellent characteristics for the treatment of AR.

## Introduction

Allergic rhinitis (AR) is a persistent, inflammatory disease characterized by anterior or posterior rhinorrhoea, nasal itching, sneezing, and nasal congestion which reduces people’s quality of life and work efficiency (Seidman et al., 2015; Brożek et al., 2017). Currently, antihistamines are the main clinical drugs for AR. However, long-term application of these drugs causes drug-induced rhinitis. Some drugs are not suitable for children and adolescents. Budesonide suspension combined with terbutaline is less effective in treating asthma at night, and the symptoms easily return (Wang et al., 2019).

Astragalus polysaccharides (APS), a main active extract of *Astragalus membranaceus*, can enhance the immune response by increasing the serum antibody titer and enhancing the secretion of a wide range of cytokines (Tan et al., 2017; Zhou et al., 2017). APS can also regulate the balance of Th1/Th2 cells and inhibit the expression of inflammatory factors and inflammation injury (Liu, 2017; Ren et al., 2018). APS ameliorated palmitate-induced pro-inflammatory responses through AMP-activated protein kinase (AMPK) activity (Lu et al., 2013).

Because nasal mucociliary clearance can reduce the residence time of drugs, microspheres are being considered as a new type of spherical carrier delivery system to overcome rapid mucociliary clearance and increase drug absorption (Ghori et al., 2015; Pandey and Tripathi, 2016). The chitosan (CTS) microsphere is a potential drug carrier and enhances absorption by opening tight junctions between the epithelial cells (Abdel Mouez et al., 2014). The CTS microsphere has been used as a promising carrier system for both vaccines and DNA (Islam et al., 2012).

Here, APS/CTS microspheres intended for nasal drug delivery were prepared by a spray-drying method, and characteristics of these microspheres were evaluated. We then explored the toxicity evaluation and therapeutic effects of APS/CTS microspheres on rats with AR.

## Materials and Methods

### Materials

Astragalus polysaccharides (purity = 70%) was provided by Hong Sheng Bio-products Company (Xi’an, China). CTS (deacetylation degree = 96.1%) was obtained from Hai Debei Marine Biotechnology Company (Jinan, China). Glacial acetic acid was kindly supplied by Yantai Shuangshuang Chemical Co., Ltd. (Yantai, China). Ovalbumin (OVA) and phosphate buffered solution (PBS) were obtained from Shanghai Solarbio Biotechnology Co., Ltd. (Shanghai, China). Budesonide was purchased from Jiangsu AstraZeneca Pharmaceutical Co., Ltd. (Jiangsu, China). Pentobarbital sodium was supplied by Beijing Rui Ee Xin De Technology Co., Ltd. (Beijing, China). The DAB kit, Biotinylated goat anti-rabbit IgG kit, and interleukin-4 (IL-4) rabbit anti-rat kit were obtained from Wuhan Boster Biological Technology Co., Ltd. (Wuhan, China). All other chemicals and solvents were analytical reagent grade.

### Preparation and Optimization of APS/CTS Microspheres

A predetermined amount of APS (2.831 g) and CTS (5 g) was dissolved in 1,000 mL of 0.5% acetic acid and stirred vigorously for 12 h under room temperature. The solution was filtrated by 0.45 μm micropore film and spray dried by a spray drier (Mini Spray-Dryer, B-290; Büchi Labortechnik AG, Flawil, Switzerland). The optimum parameters for the formulation of APS/CTS microspheres were determined using an L_9_ (3^4^) orthogonal design with three levels of four factors: inlet temperature (120, 140, 160°C), feeding rate (3, 5, 7 mL/min), molecular weight of CTS (3.0 × 10^2^, 5.0 × 10^2^, and 1.3 × 10^3^ KDa), and APS/CTS ratio (1:3, 1:5, 1:7). The exact compositions and processing parameters of individual samples were summarized in Supplementary Table 1. Subsequently, microspheres were collected and stored in desiccators for further analysis.

### Characterization of APS/CTS Microspheres

The shape and surface morphology of microspheres were observed by a scanning electron microscope (SEM) (s4500n, Hitachi, Japan). The particle size was determined by a laser diffraction particle size analyzer (Malvern Mastersizer 2000, Malvern Instruments Ltd., United Kingdom). The sample was scanned for percent transmittance in the range of 4,000–500 cm^–1^ by Fourier transform infrared spectroscopy (FTIR) (Perkin-Elmer, Japan). The thermal behaviors of pure APS, CTS and APS/CTS microspheres were determined using differential scanning calorimetry 7020 (DSC 7020) (Hitachi High-tech Science Corporation, Japan). The DSC runs were conducted over a temperature range 25–300°C at a rate of 10°C/min under inert nitrogen atmosphere.

### Determination of Yield (YD), Drug Loading (DL), and Encapsulation Efficiency (EE) of APS/CTS Microspheres

Spray-dried microspheres were dissolved in PBS, rotated for 30 min at room temperature, followed by filtering through a 0.45 μm micropore film. The supernatant was then assayed by Ultraviolet spectrophotometry (UV–vis with UV-8000A Spectrophotometer; Shanghai Puxi Instrument Factory, Shanghai, People’s Republic of China) at 490 nm. The concentration of APS was calculated according to the standard curve equation (Liu et al., 2017). Each sample was assayed in triplicate. YD, DL, and EE were calculated:

(1) $$\text{YD}\left(\%\right)=\frac{C_{M}}{C_{S}}\times 100\%$$

(2) $$\text{DL}\left(\%\right)=\frac{C_{D}}{C_{M}}\times 100\%$$

(3) $$\text{EE}\left(\%\right)=\frac{C_{E}}{C_{T}}\times 100\%$$

where *C*_*M*_ refers to the total mass of microspheres, *C*_*S*_ refers to total added solid content mass, *C*_*D*_ refers to the mass of APS in the microspheres, *C*_*E*_ refers to the mass of APS encapsulated in the microspheres, and *C*_*T*_ refers to total added APS mass.

### *In vitro* APS Release Studies

The microspheres (25 mg) were suspended in 1 mL of release medium (PBS of pH 6.8) in a dialysis bag and shaken at 100 rpm/min at 37°C. At predetermined time intervals, 1 mL of release medium was withdrawn and replaced with fresh PBS. The amount of APS was measured at 490 nm with an UV–vis. The cumulative release (CR) of APS was calculated using the equation below [13].

(4) $$\text{CR}\left(\%\right)=\sum_{t=0}^{t=\infty }\frac{M_{t}}{M_{0}}\times 100\%$$

where *M*_*t*_ refers to the cumulative releases of APS in PBS (pH 6.8) at set time intervals, and *M*_0_ refers to the APS content of APS/CTS microspheres.

### Moisture Uptake and Moisture Content

The microspheres (20 mg, *W*_*d*_) were dried to constant weight under vacuum and stored in desiccators at 40°C and 75% relative humidity (Zhang et al., 2008). The weights of microspheres (*W*_*h*_) were recorded at the end of different predetermined intervals (0.5, 1, 2, 4, 6, 8, 12, and 24 h). The increase of weight represented the amount of moisture uptake. For moisture content, the samples (20 mg) were tested at a drying temperature of 105°C for 3 h until a constant weight was achieved. Weighing was performed using a digital balance in duplicate, and the moisture absorption rate and moisture content were calculated as below (Liu et al., 2017).

(5) $$\mathrm{Moisture}\mathrm{absorption}\mathrm{rate}\left(\%\right)=\frac{W_{h}-W_{d}}{W_{\text{h}}}\times 100\%$$

(6) $$\mathrm{Moisture}\mathrm{content}\left(\%\right)=\frac{W-W^{\prime }}{W}\times 100\%$$

where *W* refers to the mass of microspheres before tested, and *W’* refers to the mass of samples after drying until the weight difference is less than 0.3 mg in two successive measurements.

### Swelling Ratio and Tap Density

The swelling behavior of microspheres was determined by assessing the water uptake. The dried microspheres were weighed (*W*_*d*_) and immersed in 4 mL of PBS (pH 6.8). The resultant mixture was incubated for 12 h to achieve swelling equilibrium. Then the solution was centrifuged at 3,000 r/min for 30 min, the liquid on the surface of microspheres was removed, and the wetted microspheres were weighed immediately (*W*_*s*_). The swelling ratio of microspheres was calculated as below (Liu et al., 2017).

(7) $$\mathrm{Swelling}\mathrm{ratio}\left(\%\right)=\frac{W_{s}-W_{d}}{W_{d}}\times 100\%$$

where *W*_*s*_ represents the weight of swollen microspheres, and *W*_*d*_ represents the weight of dried microspheres.

The bulk density of the microspheres was determined by tap density measurements (Fiegel et al., 2004). Briefly, the microspheres (25–35 mg) were loaded into a glass tube, which was tapped from a predetermined height (14 mm) on a hard bench top for 20 times. The last constant volume of the microspheres was recorded. The density was then calculated as below (Liu et al., 2017).

(8) $$\rho =\frac{m}{V}$$

where ρ refers to the tap density, *m* refers to the recorded mass of the microspheres, and *V* refers last constant volume of the microspheres.

### Preparation of AR Model Rats and Administration of Test Drugs

All animal procedures were conducted in accordance with the US NIH Guidelines for the Care and Use of Laboratory Animals, and the animal study was approved by the Animal Ethical Committee of Weifang Medical University. A total of 60 healthy Wistar rats (female, 5–6 weeks old, 160–200 g, Shandong Lukang Animal Center, license no., Slxklu no. 2015002; Shandong, China) were obtained. Rats were randomly divided into six groups: the normal group, model group, budesonide group (20 μg/kg), APS/CTS microspheres (low-dose, 5 mg/kg) group, APS/CTS microspheres (medium-dose, 10 mg/kg) group, and the APS/CTS microspheres (high-dose, 15 mg/kg) group. In the sensitization phase, OVA (0.3 mg/mL) with aluminum hydroxide gel (30 mg) were administered to rats by intraperitoneal injection once every other day for a total of 14 days (Daoud et al., 2014). In the challenge phase (day 15–21), bilateral nasal cavities of rats were continuously instilled by 50 μL OVA (25 mg/mL) once a day. Sneezing and scratching were observed within 30 min after each nasal instillation, and rats were then administrated. Rats were administrated from day 22 to day 32. In the model group and normal group, rats were administrated with only sterile normal saline once a day. In the budesonide group, rats were administrated with budesonide at a dose of 20 μg/kg once a day. In the APS/CTS microspheres low, medium, and high-dose group, rats were separately administrated with APS/CTS microspheres at a dose of 5, 10, 15 mg/kg (APS/CTS microspheres suspended in normal saline and nasal dripped with pipette tips) once a day. Except for the normal group, rats of other groups maintained the OVA nasal drops once every other day and nasal administration was performed 20 min before each challenge.

### Allergic Symptoms Evaluation of Rats

The allergic symptoms of rats were observed and scored. The rats were observed for sneezing, scratching, and rhinorrhea and scored within 30 min after each challenge or administration. The allergic rat model was considered to be successful when symptom scores were higher than 5 (Senturk et al., 2018). Score 1: the time of sneezing was 1–3 or scratching was 1–5 or reaching the anterior nostril; Score 2: the time of sneezing was 4–10 or scratching was 6–15 or over the anterior nostril; and Score 3: the time of sneezing was more than 10 or scratching was more than 15 or flowing all the face.

### Eosinophil and Neutrophil Counts in Nasal Lavage Fluid

At 24 h after the last nasal drop, rats in each group were anesthetized with 0.3% sodium pentobarbital (40 mg/kg). A syringe (1 mL) was inserted into the nasal cavity of rats about 1.5 cm and was fixed, the frog wood anatomy board and rats were inverted, 3 mL sterile normal saline (1 mL/min) was slowly injected into one side of nasal cavity, and lavage fluid was recovered in the other side. The nasal lavage fluid was centrifuged for 10 min at 4°C, 1,500 rpm/min, and the cell pellet was taken to count the eosinophils and neutrophils (Virtala et al., 2012).

### Hematoxylin-Eosin (HE) Staining and Immunohistochemistry

These nasal mucosa tissue specimens and the livers, kidneys, and spleens of rats were fixed in 4% paraformaldehyde for 24 h and cut into suitable size, placed in the embedding box, dehydrated by a serial alcohol gradient, embedded in paraffin wax, and cooled in a refrigerator at 4°C to solidify. Nasal mucosa tissue and viscera specimens were cut into 4 μm thick slices, and these were then dewaxed in xylene, rehydrated by different concentrations of ethanol, washed in distilled water, and stained with HE. These slides were dehydrated with ethanol, incubated in 3% hydrogen peroxide methanol, and then incubated with IL-4 polyclonal antibody (1:100 dilution) overnight at 4°C. This was followed incubation with the horseradish peroxidase labeled secondary antibody. The slides were then stained with DAB kit.

### Statistical Analysis

All experiments were performed three times, and the results were reported as mean ± standard deviations. Statistical analysis was carried out using SPSS19.0 and all statistically significant differences were analyzed by one-way analysis of variance and Newman–Keuls test. In all cases, *p* < 0.05 was considered statistically significant.

## Results

### Morphology and Particle Size

**FIGURE 1:**
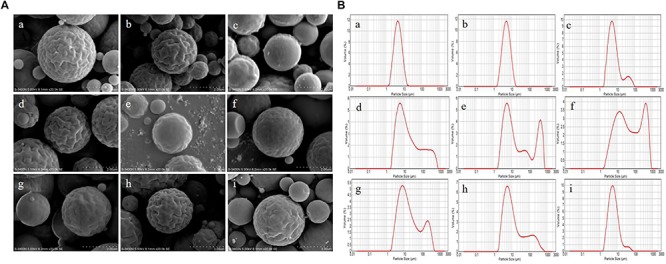
Morphology and particle size of Astragalus polysaccharides/chitosan microspheres. APS, Astragalus polysaccharides; CTS, chitosan; Inlet temperature; feeding rate; molecular weight of CTS and APS/CTS ratio: (a) 120°C, 3 mL/min, 300 KDa, 1:3; (b) 120°C, 5 mL/min, 500 KDa, 1:5; (c) 120°C, 7 mL/min, 1300 KDa, 1:7; (d) 140°C, 3 mL/min, 500 KDa, 1:7; (e) 140°C, 5 mL/min, 1300 KDa, 1:3; (f) 140°C, 7 mL/min, 300 KDa, 1:5; (g) 160°C, 3 mL/min, 1300 KDa, 1:5; (h) 160°C, 5 mL/min, 300 KDa, 1:7; (i) 160°C, 7 mL/min, 500 KDa, 1:3. **(A)** SEM of spray-dried APS/CTS microspheres. **(B)** Particle-size distribution of APS/CTS microspheres.

The optimization of experimental conditions was performed with regard to the DL. As shown in Supplementary Table 2, the optimal formulation was drawn: inlet temperature of 120°C, feeding rate of 3 mL/min, molecular weight of CTS of 5.0 × 10^2^ KDa, and APS/CTS ratio of 1:3. The morphology of APS/CTS microspheres was confirmed via SEM imaging, as demonstrated in Figure 1A. The microspheres in every group displayed regular spherical shape and were presented in different sizes and different extents of surface roughness. The microsphere of a, b, d, and h presented considerably wrinkled surfaces; slightly wrinkled surfaces were found in microsphere e, f, g, and i; and microsphere c displayed a smooth surface. Adhesion phenomena were found in microsphere a, b, and c.

The volume size distributions of microspheres were shown in Figure 1B. The volume distribution of 50% [d (0.5)] of microsphere a, b c, d, e, f, g, h, and i was 4.74, 5.28, 5.94, 11.97, 12.37, 42.02, 13.99, 8.88, and 5.68 μm, respectively. Among them, microsphere a, b, and i displayed narrow particle size distribution, and the other microspheres indicated that their size distributions were bimodal.

### Results of FTIR and DSC

**FIGURE 2:**
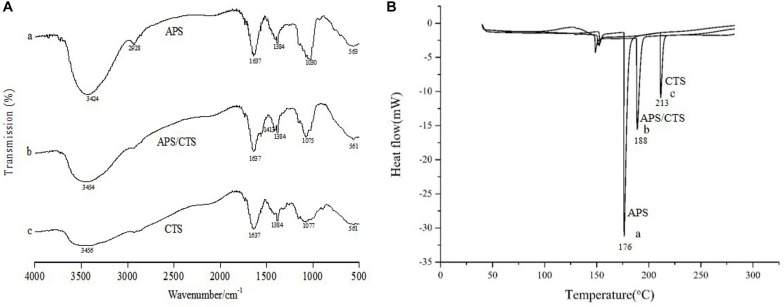
FTIR and DSC of Astragalus polysaccharides/chitosan microspheres. APS, Astragalus polysaccharides; CTS, chitosan; **(A)** FTIR spectra of APS/CTS microspheres. (a) APS, (b) APS/CTS microspheres, and (c) CTS. **(B)** DSC thermograms of APS/CTS microspheres. (a) APS, (b) APS/CTS microspheres, and (c) CTS.

In Figure 2A, FTIR showed that spectra of pure CTS had a broad absorption band at 3,456 cm^–1^ (O-H stretching vibration) and the characteristic absorptions of CTS were the bands at 1,637 cm^–1^ (amide I, C-O stretching mode conjugated with -N-H deformation mode), at 1,384 cm^–1^ (C-O-C stretching vibration) and at 1,077 cm^–1^ (skeletal vibration involving C-O stretching). The FTIR spectra showed that the absorption peaks of APS were at 3,424 cm^–1^ (O-H stretching vibration), which reflected the -inter and -intra molecular interactions of the polysaccharide, the absorptions at 2,928 cm^–1^ (C-H stretching vibration) and 1,637 cm^–1^ (COO^–^ stretching vibration). Compared with APS and CTS, the spectra of the APS/CTS microspheres showed a slight shift at 3,454 and 1,075 cm^–1^. The change in the curve of the APS/CTS microspheres indicated that APS could react with CTS (Jiang et al., 2013; Liu et al., 2017; Liao et al., 2018). The DSC thermograms of APS, CTS, and APS/CTS microspheres were shown in Figure 2B. A sharp endothermic peak of APS was at 176°C. The thermogram of CTS exhibited an endothermic peak at 213°C. For APS/CTS microspheres, a melting peak was observed at 188°C. These results indicated that APS and CTS had the properties of crystals. The DSC of the APS-loaded microspheres indicated a sharp endothermal peak at 188°C where no characteristic peak of APS was observed. The disappearance of the drug peak revealed that APS was entrapped or partially entrapped in the APS/CTS microspheres.

### Characteristics of Microspheres

**FIGURE 3:**
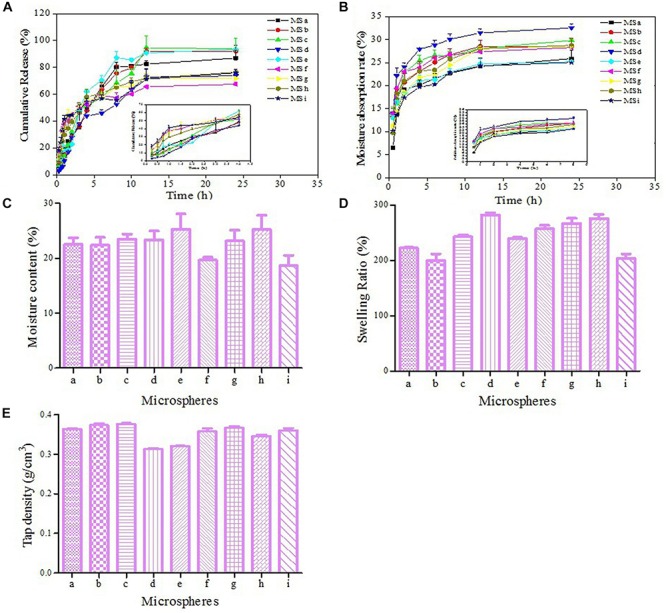
Characteristics of microspheres. APS, Astragalus polysaccharides; CTS, chitosan; Inlet temperature; feeding rate; molecular weight of CTS and APS/CTS ratio: (a) 120°C, 3 mL/min, 300 KDa, 1:3; (b) 120°C, 5 mL/min, 500 KDa, 1:5; (c) 120°C, 7 mL/min, 1300 KDa, 1:7; (d) 140°C, 3 mL/min, 500 KDa, 1:7; (e) 140°C, 5 mL/min, 1300 KDa, 1:3; (f) 140°C, 7 mL/min, 300 KDa, 1:5; (g) 160°C, 3 mL/min, 1300 KDa, 1:5; (h) 160°C, 5 mL/min, 300 KDa, 1:7; (i) 160°C, 7 mL/min, 500 KDa, 1:3. **(A)** *In vitro* release profile of APS from APS/CTS microspheres at pH 6.8 under 37°C (mean ± SD, *n* = 3). **(B)** The moisture absorption rate of APS/CTS microspheres (mean ± SD, *n* = 3). **(C)** The moisture content of APS/CTS microspheres (mean ± SD, *n* = 3). **(D)** Swelling ratio of APS/CTS microspheres (mean ± SD, *n* = 3). **(E)** Tap density of APS/CTS microspheres (mean ± SD, *n* = 3).

The cumulative release curves of APS were shown in Figure 3A and Supplementary Table 3. An obvious burst effect at the first 2 h was observed, and 67.48–93.76% APS was steadily released from APS/CTS microspheres at pH 6.8 within 24 h. Moisture absorption rates of all microspheres at 40°C and RH 75% were displayed in Figure 3B, and these were from 6.49 to 13.97% within 0.5 h. Microsphere i had the lowest moisture absorption rate at 12th h. Figure 3C showed that the moisture contents of all microspheres ranged from 18.59 to 25.28%. The swelling ratios of APS/CTS microspheres were revealed in Figure 3D and Supplementary Table 3, and they were from 200.22 to 282.22%. Figure 3E displayed tap densities of spray-dried microspheres ranged from 0.31 to 0.38 g/cm^3^.

### Evaluation of Symptom After Nasal Administration

**FIGURE 4:**
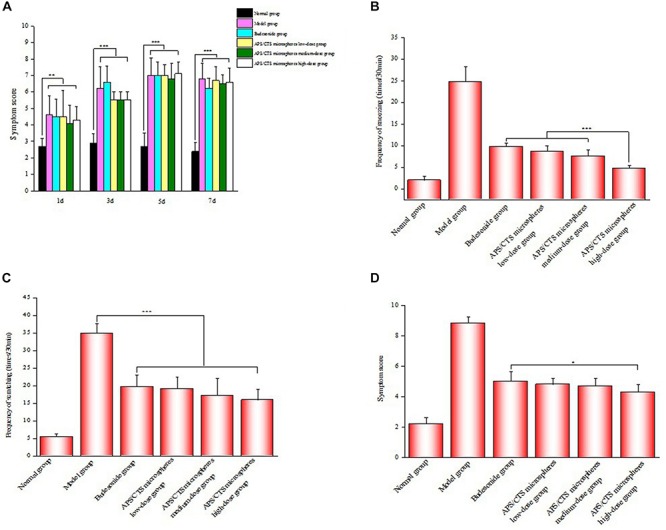
Evaluation of symptom after nasal administration. **(A)** Results of symptom score evaluation in molding stage (mean ± SD, *n* = 10, **p < 0.01, ***p < 0.001). **(B)** Frequency of sneezing of rats after nasal administration (mean ± SD, *n* = 10, ***p < 0.001). **(C)** Frequency of scratching of rats after nasal administration (mean ± SD, *n* = 10, ***p < 0.001). **(D)** Results of symptom score evaluation after nasal administration (mean ± SD, *n* = 10, *p < 0.05).

The allergic symptoms of rats were scored on the 1st, 3rd, 5th, and 7th day of challenge phase (Figure 4A). There was a significant difference between APS/CTS microspheres (high-dose) group and budesonide group concerning the frequency of the sneezing of rats (Figure 4B). Figure 4C shows an evident difference between the budesonide group, the APS/CTS microsphere (low, medium, and high-dose) group, and the model group regarding the frequency of scratching in rats. In Figure 4D, an obvious difference between the APS/CTS microsphere (high-dose) group and the budesonide group about symptom score evaluation after nasal administration could be seen.

### Eosinophils and Neutrophils Count in Nasal Lavage Fluid and Morphological Change of Nasal Mucosa

**FIGURE 5:**
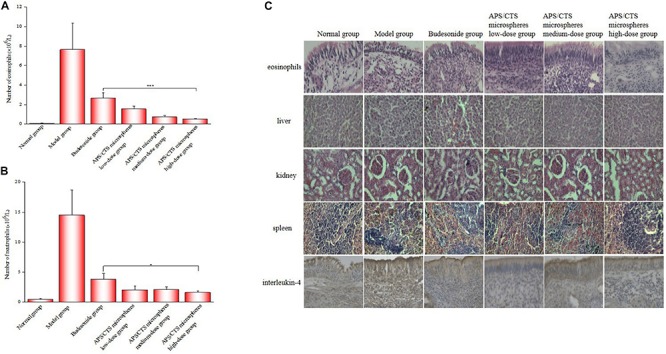
Eosinophil and neutrophil count in nasal lavage fluid and morphological change of nasal mucosa. **(A)** Number of eosinophils in nasal lavage fluid (mean ± SD, *n* = 10, ***p < 0.001). **(B)** Number of neutrophils in nasal lavage fluid (mean ± SD, *n* = 10, *p < 0.05). **(C)** HE staining of eosinophils, liver, kidney and spleen and immunohistochemical staining of IL-4 in nasal mucosa tissue.

As was shown in Figures 5A,B, the APS/CTS microsphere (high-dose) group exhibited a lower number of eosinophils and neutrophils in nasal lavage fluid compared to the budesonide group. In Figure 5C, a large number of eosinophils could be seen to have infiltrated into the subepithelial lamina propria, and there were submucosal vasodilation, congestion, and tissue edema in the model group; the nasal mucosa of the APS/CTS microsphere (high-dose) group displayed less infiltration of eosinophils compared to budesonide group. The structures of the liver, kidneys, and spleen treated with microspheres were normal. Compared to budesonide group, the expression of IL-4 in APS/CTS microspheres (medium, high-dose) group was significantly reduced.

## Discussion

Allergic rhinitis is a chronic inflammatory disease of the upper respiratory tract resulting from various environmental factors. APS can effectively correct the imbalance of Th1/Th2 cells and reduce inflammatory infiltration, and it has been widely used in the clinical treatment of AR (Liu et al., 2017; Ren et al., 2018). CTS microspheres can overcome rapid mucociliary clearance and provide longer contact time for drug transport. For example, Pandey et al. prepared and optimized CTS microspheres loaded with levocetirizine for nasal delivery with the aim to prolong residence time, increase local absorption of the drug, and improve therapeutic effects. Therefore, CTS microspheres have been extensively evaluated as a drug delivery system (Pandey and Tripathi, 2016).

In our study, APS/CTS microspheres were prepared by the spray-drying method to treat rats with AR. APS/CTS microspheres showed wrinkled surfaces, which could enable them adhere to nasal mucosal epithelial cells and prolong the drug treatment time (Liu et al., 2017). The rate of the heat transfer and water diffusion from the surface to the core of microspheres, as well as the intermediate air inlet temperature and feed flow rate, consequently influenced the microstructure of spray-dried microspheres (Behboudi-Jobbehdar et al., 2013). The size of microsphere f was 42.02 μm, and it could be administered through the nasal cavity. The DSC confirmed that APS was entrapped or partially entrapped in the APS/CTS microspheres. In terms of *in vitro* release, there was an obvious burst effect of all microspheres at the first 2 h, and 67.48–93.76% of the APS was then steadily released from APS/CTS microspheres at pH 6.8 within 24 h, which indicated that the ASP/CTS microspheres were ideal for sustained-release preparations, which have the potential to prolong the action time of the drug in nasal mucosa, reduce the frequency of taking medicine, and improve patients compliance. The obvious burst effect was due to the release of APS adsorbed on the surface, and the sustained release was due to the dissolution of CTS (Zhang et al., 2007). Yu et al. (Yu et al., 2018) studied the release of proanthocyanidin from CTS microspheres, and the *in vitro* release study showed that 76.92% of proanthocyanidin was released from microspheres within 48 h. Therefore, the type of drug and the ratio of drug to the carrier will affect the release of the drug in the carrier.

Furthermore, the OVA-induce AR rat model was used to evaluate the effect of APS/CTS microspheres. Results revealed that, compared to budesonide group, symptom scores of rats significantly reduced in APS/CTS microspheres group. Eosinophils can invade the respiratory epithelium and produce a number of toxic mediators that may be responsible for some of the epithelial hyperplasia, hyalinosis, and damage (Johnson et al., 2007; Cho et al., 2015). Our results indicated that APS/CTS microsphere administration groups evidently reduced the infiltration of eosinophils and relieved submucosal vasodilatation and tissue edema. T lymphocytes are pivotal immune cells in allergic responses, especially Th1 and Th2. Th2 cells are characterized to secrete IL-4, which induces the production of IgE and contributes to the development of allergic inflammation (Liu et al., 2017; Shao et al., 2017). The positive signal of IL-4 in the nasal mucosa of the APS/CTS microsphere administration groups was significantly lower than that of the model group and budesonide group.

According to some studies (Senturk et al., 2018), cyclosporine nasal sprays have been used to treat AR in an animal model (54 healthy female Sprague-Dawley rats). In our study, APS/CTS microspheres were designed for nasal delivery in a total of 60 healthy Wistar rats. In the biochemical examination, Erol Senturk et al. (2018) studied the surface of the tissue tumor necrosis factor (TNF), interferon (IFN), interleukin (IL)-5, IL-13, IL-2, IL-4, IL-17A, and IgE. In a future experiment, we will further explore these responses and their application in human AR.

## Conclusion

In this research, APS/CTS microspheres intended for nasal drug delivery were successfully prepared by the spray-drying method. The APS/CTS microspheres exhibited positive characteristics, such as uniform morphology, a particle size of 40–60 μm, which be suitable for nasal administration, and an excellent DL capacity of 9.11–21.50%. The DSC indicated that the APS was entrapped or partially entrapped in the APS/CTS microspheres. Furthermore, *in vitro* release revealed the potential of microspheres for extended release of APS. APS/CTS microspheres could alleviate allergic symptoms as well as reduce eosinophils infiltration and the expression of IL-4 in nasal mucosa of rats. In addition, APS/CTS microspheres did not display liver and kidney toxicity through HE-staining observation. Thus, our study has suggested that spray drying could serve as a simple and efficient method for preparing APS/CTS microspheres, which had excellent characteristics, therapeutic effects on rats with AR, and a promising potential for sustained local drug delivery. However, the study had some other limitations, and the next experimental research will be conducted by increasing sample size and studying other APS/CTS microsphere properties and how they should be applied to AR in humans.

## Data Availability Statement

The datasets generated for this study are available on request to the corresponding author.

## Ethics Statement

All animal procedures were conducted in accordance with the US NIH Guidelines for the Care and Use of Laboratory Animals, and the animal study was approved by the Animal Ethical Committee of the Weifang Medical University.

## Author Contributions

SW prepared the APS/CTS microspheres by the spray-drying method, treated the AR of rats with them, and completed the yield, drug loading, and encapsulation efficiency of microspheres. YS performed the *in vitro* APS release studies and completed the moisture uptake and moisture content of microspheres. JZ performed the characterization of microspheres. XC, ZX, and DD performed the study of the swelling ratio and tap density of microspheres. LZ, WL, and WZ directed and planned the entire experiment.

## Conflict of Interest

The authors declare that the research was conducted in the absence of any commercial or financial relationships that could be construed as a potential conflict of interest.
