# Supplementary material for: Astragalus Polysaccharides/Chitosan Microspheres for Nasal Delivery: Preparation, Optimization, Characterization, and Pharmacodynamics
Source: Front Pharmacol. 2020 Mar 18;11:230. doi: 10.3389/fphar.2020.00230 (PMC7093564; doi:10.3389/fphar.2020.00230)
Supplement: Supplementary file 1 [file Data_Sheet_1.docx]

**Supplemental Table 1** The factors and levels discussed in the orthogonal design.

| **Levels** | **Factors** | | | |
| --- | --- | --- | --- | --- |
|  | **A (°C)** | **B (mL/min)** | **C (KDa)** | **D (w/w)** |
| 1 | 120 | 3 | 300 | 1:3 |
| 2 | 140 | 5 | 500 | 1:5 |
| 3 | 160 | 7 | 1300 | 1:7 |

(A) Inlet temperature; (B) feeding rate; (C) molecular weight of chitosan; (D) Astragalus polysaccharides/chitosan ratio.

**Supplemental Table 2** Yield, drug loading and encapsulation efficiency of Astragalus polysaccharides/chitosan microspheres (Mean±Standard deviation, n=3).

| **MS** | **A (°C)** | **B**  **(mL/min)** | **C (KDa)** | **D**  **(w/w)** | **YD**  **(%)** | **DL**  **(%)** | **EE**  **(%)** |
| --- | --- | --- | --- | --- | --- | --- | --- |
| a | 120 | 3 | 300 | 1:3 | 81.93 | 21.50±0.01 | 95.21±0.04 |
| b | 120 | 5 | 500 | 1:5 | 74.13 | 15.14±0.02 | 97.30±0.12 |
| c | 120 | 7 | 1300 | 1:7 | 48.71 | 11.03±0.01 | 92.96±0.10 |
| d | 140 | 3 | 500 | 1:7 | 60.79 | 9.98±0.004 | 84.11±0.03 |
| e | 140 | 5 | 1300 | 1:3 | 38.57 | 12.27±0.05 | 64.33±0.10 |
| f | 140 | 7 | 300 | 1:5 | 62.32 | 12.65±0.01 | 81.26±0.02 |
| g | 160 | 3 | 1300 | 1:5 | 59.26 | 12.42±0.01 | 79.86±0.02 |
| h | 160 | 5 | 300 | 1:7 | 64.88 | 9.11±0.02 | 76.83±0.02 |
| i | 160 | 7 | 500 | 1:3 | 73.27 | 18.73±0.04 | 77.94±0.11 |

(A) inlet temperature; (B) feeding rate; (C) molecular weight of CTS; (D) APS/CTS ratio. Inlet temperature; feeding rate; molecular weight of CTS and APS/CTS ratio: (a) 120 ℃, 3 mL/min, 300 KDa, 1:3; (b) 120 ℃, 5 mL/min, 500 KDa, 1:5; (c) 120 ℃, 7 mL/min, 1300 KDa, 1:7; (d) 140 ℃, 3 mL/min, 500 KDa, 1:7; (e) 140 ℃, 5 mL/min, 1300 KDa, 1:3; (f) 140 ℃, 7 mL/min, 300 KDa, 1:5; (g) 160 ℃, 3 mL/min, 1300 KDa, 1:5; (h) 160 ℃, 5 mL/min, 300 KDa, 1:7; (i) 160 ℃, 7 mL/min, 500 KDa, 1:3. Abbreviations: APS, Astragalus polysaccharides; CTS, chitosan; MS, microsphere; YD, yield; DL, drug loading; EE, encapsulation efficiency.

**Supplemental Table 3** Swelling ratio and *in vitro* release of microspheres.

| **MS** | **Swelling ratio (%)** | ***In vitro* release (%)** | |
| --- | --- | --- | --- |
|  |  | **the first 2 hours** | **24 hours** |
| a | 222.2±1.26 | 30.25±0.33 | 86.86±5.84 |
| b | 200.22±11.46 | 28.37±0.39 | 92.07±4.35 |
| c | 242.72±3.03 | 32.15±0.34 | 93.76±8.18 |
| d | 282.22±3.59 | 27.6±2.96 | 73.7±4.79 |
| e | 239.83±1.71 | 22.81±0.60 | 93.37±2.71 |
| f | 257.20±5.90 | 44.86±0.37 | 67.48±1.58 |
| g | 266.85±8.90 | 44.57±0.50 | 71.71±1.03 |
| h | 275.65±7.28 | 39.50±2.07 | 76.52±2.15 |
| i | 203.55±7.75 | 44.54±0.87 | 75.93±2.73 |

Inlet temperature; feeding rate; molecular weight of CTS and APS/CTS ratio: (a) 120 ℃, 3 mL/min, 300 KDa, 1:3; (b) 120 ℃, 5 mL/min, 500 KDa, 1:5; (c) 120 ℃, 7 mL/min, 1300 KDa, 1:7; (d) 140 ℃, 3 mL/min, 500 KDa, 1:7; (e) 140 ℃, 5 mL/min, 1300 KDa, 1:3; (f) 140 ℃, 7 mL/min, 300 KDa, 1:5; (g) 160 ℃, 3 mL/min, 1300 KDa, 1:5; (h) 160 ℃, 5 mL/min, 300 KDa, 1:7; (i) 160 ℃, 7 mL/min, 500 KDa, 1:3. Abbreviations: MS, microsphere.
